# (−)-Epigallocatechin-3-Gallate Induces Non-Apoptotic Cell Death in Human Cancer Cells via ROS-Mediated Lysosomal Membrane Permeabilization

**DOI:** 10.1371/journal.pone.0046749

**Published:** 2012-10-08

**Authors:** Yin Zhang, Nai-Di Yang, Fan Zhou, Ting Shen, Ting Duan, Jing Zhou, Yin Shi, Xin-Qiang Zhu, Han-Ming Shen

**Affiliations:** 1 Department of Toxicology, Zhejiang University School of Public Health, Hangzhou, Zhejiang, China; 2 Department of Physiology, Yong Loo Lin School of Medicine, National University of Singapore, Singapore, Singapore; 3 Saw Swee Hock School of Public Health, National University of Singapore, Singapore, Singapore; 4 NUS Graduate School for Integrative Sciences and Engineering, National University of Singapore, Singapore, Singapore; Emory University, United States of America

## Abstract

(−)-Epigallocatechin-3-gallate (EGCG) is the most extensive studied tea polyphenol for its anti-cancer function. In this study, we report a novel mechanism of action for EGCG-mediated cell death by identifying the critical role of lysosomal membrane permeabilization (LMP). First, EGCG-induced cell death in human cancer cells (both HepG2 and HeLa) was found to be caspase-independent and accompanied by evident cytosolic vacuolization, only observable when cells were treated in serum-free medium. The cytosolic vacuolization observed in EGCG-treated cells was most probably caused by lysosomal dilation. Interestingly, EGCG was able to disrupt autophagic flux at the degradation stage by impairment of lysosomal function, and EGCG-induced cell death was independent of Atg5 or autophagy. The key finding of this study is that EGCG is able to trigger LMP, as evidenced by Lyso-Tracker Red staining, cathepsin D cytosolic translocation and cytosolic acidification. Consistently, a lysosomotropic agent, chloroquine, effectively rescues the cell death via suppressing LMP-caused cytosolic acidification. Lastly, we found that EGCG promotes production of intracellular ROS upstream of LMP and cell death, as evidenced by increased level of ROS in cells treated with EGCG and the protective effects of antioxidant N-acetylcysteine (NAC) against EGCG-mediated LMP and cell death. Taken together, data from our study reveal a novel mechanism underlying EGCG-induced cell death involving ROS and LMP. Therefore, understanding this lysosome-associated cell death pathway shed new lights on the anti-cancer effects of EGCG.

## Introduction

The benefits of tea consumption to health have been well established via various studies in humans. Most of such benefits, including prevention of cancer and cardiovascular diseases, have been attributed to the polyphenolic components in tea [1]. As the most abundant and biologically active constituent among the tea polyphenols, (−)-epigallocatechin-3-gallate (EGCG) has received a great deal of attention in cancer research [2]. To date, the mechanisms underlying the anti-cancer function of EGCG have been studied extensively. It is known that EGCG can bind to multiple molecular targets, including transmembrane receptors, kinases or other key proteins, thus affects a range of signaling pathways, resulting in growth inhibition, apoptosis or suppression of invasion, angiogenesis and metastasis [3]. Among them, the ability of EGCG for induction of cell death in cancer cells is considered as one of the key mechanisms related to its anti-cancer function [4]. Nevertheless, the exact molecular mechanisms for EGCG-induced cell death have not been fully elucidated. Most of previous reports have concluded that EGCG induces caspase-mediated apoptosis in various tumor cells via mitochondrial pathway [5], [6] or via the death receptor [7], while only a few studies demonstrated non-apoptosis cell death caused by EGCG [8], [9].

Among various mechanisms for EGCG-mediated cell death, reactive oxygen species (ROS) and oxidative stress appears to be particularly important. Although EGCG with a pyrogallol-type structure on the B-ring processes a strong antioxidant activity, this structure is proved to be unstable in cell cultured system [10]. The auto-oxidation of EGCG in Dulbecco's modified Eagle's medium (DMEM) produces substantial amount of ROS, especially H_2_O_2_, which plays an important role in the cytotoxic effect of EGCG against cancer cell lines [11], [12]. Addition of antioxidants into the culture medium was reported to inhibit EGCG-induced apoptosis [13], [14]. Recently, the involvements of ROS and oxidative stress in non-apoptotic cell death or necrosis have been increasing appreciated [15]. Therefore, it is of interest to further understand the role of ROS and oxidative stress in EGCG-mediated cell death, including both apoptotic and non-apoptotic cell death.

Lysosomes are cytoplasmic membrane-enclosed organelles that contain hydrolytic enzymes and that control the intracellular turnover/degradation of macromolecules [16]. In recent years, the biological function of lysosomes has been increasingly appreciated, and it is known to play critical roles in various physiological processes such as autophagy and in human diseases such as lysosomal storage diseases, cancer and neurodegenerative diseases [17]. Lysosomal proteases, which are held within the membrane under normal conditions, would leak into the cytosol in both apoptosis and necrosis [18]. One key process that is known to be closely associated with the cell death process is lysosomal membrane permeabilization (LMP) [19]. The exact outcome of LMP is dependent on the extent of lysosomal membrane damage. A massive rupture of lysosomes and rapid release of their acidic contents are often a critical step for necrosis; while the partial and selective lysosomal leakage is often associated with the apoptotic process [20], [21]. There are many known factors to disrupt lysosomal membrane integrity and cause LMP, including ROS, lysosomotropic detergents, microtubule toxins and some lipids [17], [19]. For instance, some anti-cancer agents such as vincristine and siramesine are known to cause lysosome-dependent cell death [22], [23]. So far, it is not known whether lysosome is implicated in cell death induced by EGCG in cancer cells.

In this study we aimed to further examine the molecular mechanisms underlying EGCG-mediated cell death. Our results first showed that EGCG induces a form of caspase-independent non-apoptotic cell death, which can be significantly augmented in the serum-free medium. Second, we found that EGCG triggers LMP, resulting in leakage of its key proteases (cathepsins) into the cytosol, and cell death. Interestingly, such cell death is independent of autophagy. Finally, we provided clear evidence showing that EGCG promotes intracellular ROS formation which functions as a key process leading to LMP and cell death. Therefore, data from this study identify a novel cell death mechanism induced by EGCG in cancer cells which involves oxidative stress, lysosomal damage and eventually cell death.

**Figure 1:**
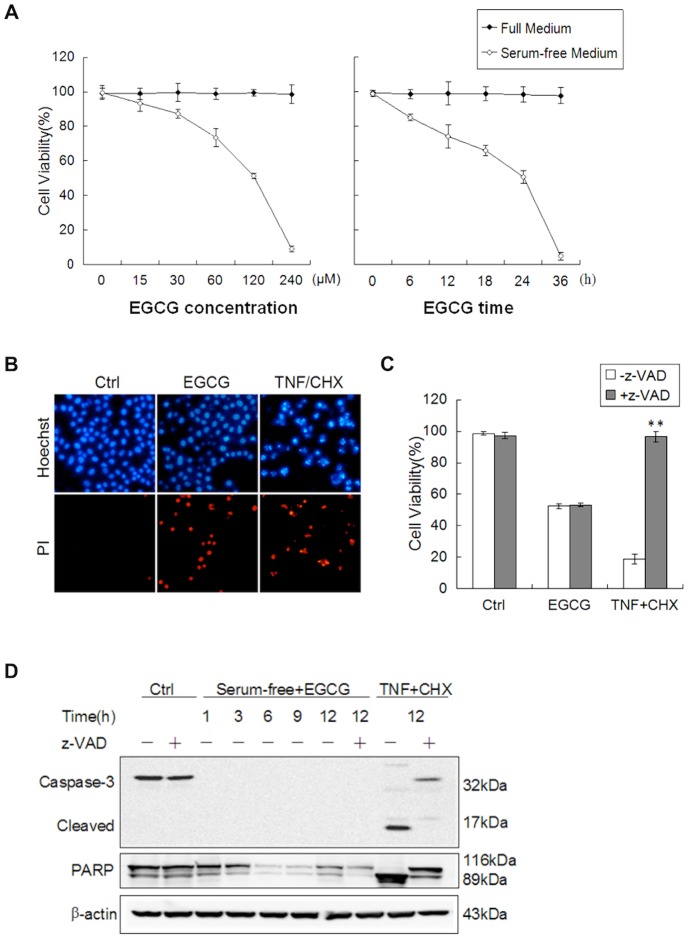
Serum starvation enhances EGCG-induced cell death independent of caspase. (A) Serum deprivation promotes EGCG-induced cell death in a concentration-dependent and time-course manner. HepG2 cells were treated with different doses of EGCG in full or serum-free medium for 12 h (left panel) or with 60 µM EGCG for different time as indicated (right panel). The cell viability was determined by Hoechst-PI double staining (n = 3, mean ± SD). (B) Representative pictures of Hoechst-PI double staining. HepG2 cells were cultured in full medium (as a control); treated with 60 µM EGCG for 12 h in serum-free medium; or incubated with 20 ng/ml TNFα and 10 µg/ml CHX for 12 h in full medium (as a positive control for apoptosis). (C) EGCG induces caspase-independent cell death. HepG2 cells were treated with EGCG (60 µM×24 h) or in the absence or presence of 40 µM z-VAD-fmk. The co-treatment with TNFα (20 ng/ml) and CHX (10 µg/ml) for 12 h was used as a positive control. Cell viability was determined as described in Panel A. **p<0.005 comparing to the group without z-VAD (Student's *t*-test, n = 3). (D) No caspase-3 activation and PARP cleavage cause by EGCG-induced cell death. Cells were treated with EGCG or TNF/CHX as described in panel C, and cell lysates were collected and subject to western blot.

## Materials and Methods

### Chemicals, Reagents, and Antibodies

(−)-Epigallocatechin-3-gallate (EGCG, >90%) was purchased from Zhejiang University Tea Research Institute. 5-(and-6)-chloromethyl-2',7'-dichlorodihydrofluorescein diacetate acetyl ester (CM-H_2_DCFDA), Hoechst33342, and Lyso-Tracker® Red (DND-99) was obtained from Invitrogen. z-VAD-fmk was form Enzo. Acridine orange was from Immunochemistry Technologies. E-64D and N-acetylcysteine were from Merk. Propidium iodide (PI), digitonin, chloroquine (CQ), bafilomycin A1 (Baf A1), pepstatin A and other common chemicals were all purchased from Sigma-Aldrich. The primary antibodies used in the study include the following: anti-poly (ADP-ribose) polymerase (PARP), anti-caspase-3 and anti-glyceraldehyde-3-phosphate dehydrogenase (GAPDH) from Cell Signaling, anti-β-actin and anti-microtubule-associated protein 1 light chain 3 (LC3) from Sigma-Aldrich, anti-p62 from Abnova, anti-Atg5, anti-cathepsin D and anti-lysosome-associated membrane protein (LAMP-1) from Santa Cruz. The secondary antibodies, horseradish peroxidase-conjugated goat anti-mouse IgG, goat anti-rabbit IgG and rabbit anti-goat IgG, were all from Thermo Scientific.

**Figure 2:**
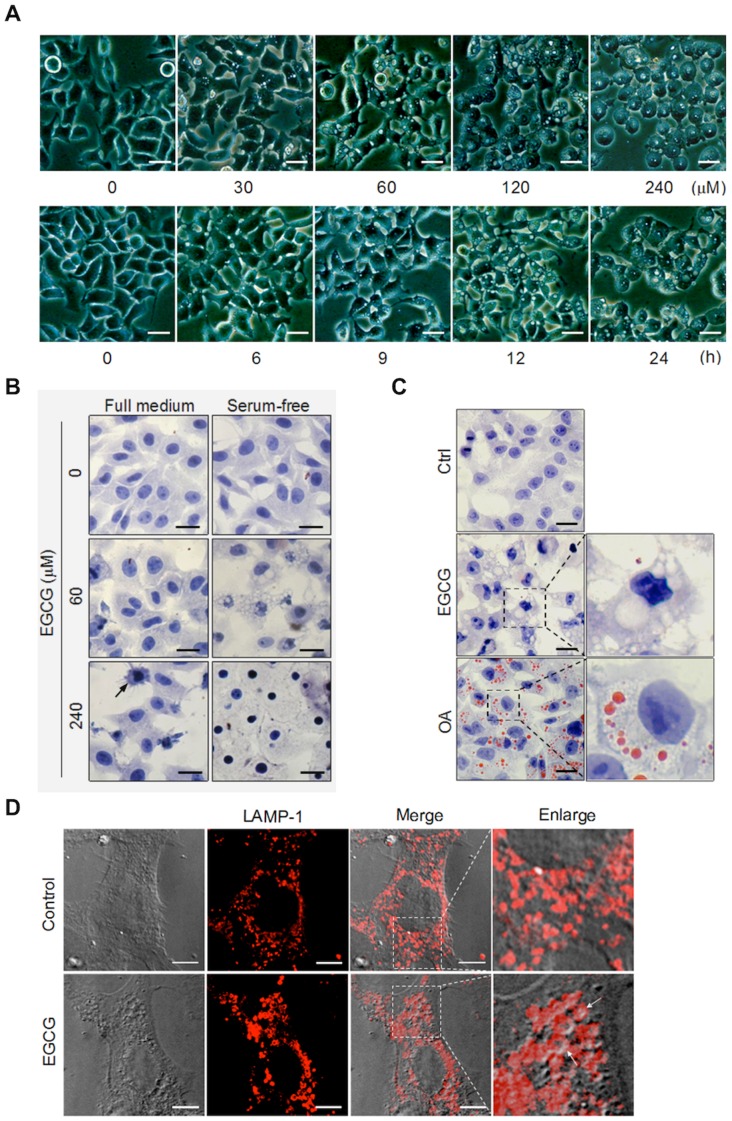
EGCG induces cytosolic vacuolization. (A) Morphological alterations of EGCG-treated cells in serum-free medium were analyzed using light microscopy. Representative pictures of HepG2 cell treated with EGCG at indicated concentrations for 12 h (upper panel) or with 60 µM EGCG for indicated time courses (lower panel) are shown (scale bar: 50 µm). (B) HepG2 cells were treated with EGCG (60 or 240 µM) for 12 h. The cells were then fixed and stained by hematoxylin, then analyzed by light microscopy (scale bar: 30 µm). (C) The vacuole contents are not lipid droplets. HepG2 cells were EGCG (60 µM) for 12 h. Cultured cells in full medium for 12 h were used as a negative control, and cells treated with 1 mM oleate acid (OA) in DMEM medium containing 1%BSA for 12 h were used as the positive control. The cells were fixed and stained with 0.5% Oil Red O and hematoxylin (scale bar: 30 µm). (D) The vacuoles are of lysosome origin. Immunofluorescence staining of LAMP-1 was performed in MEF cells after treatment with or without EGCG (60 µM) for 9 h (scale bar: 10 µm).

### Cell Culture and Treatments

Human heptoma cell line HepG2 was from the American Type Culture Collection. Mouse embryonic fibroblasts (MEF), both wild-type (WT) and m5–7 cells with inducible deletion of Atg5 were provided by Dr. N. Mizushima (Tokyo Medical and Dental University) [24]. Both of them were grown in DMEM (Sigma-Aldrich) containing 10% fetal bovine serum (HyClone) and 1% penicillin-streptomycin (Invitrogen), in a 5% CO_2_ atmosphere at 37°C.

### Cell Viability Assays

In this study, cell viability was quantified using PI exclusion test as described previously with modifications [25]. For each sample, cells were seeded into a 96-well plate at 5×10^3^ per well. The next day, cells were first treated as described in the results and in the figure legends, followed by incubation with 10 µg/ml Hoechst33342 and 5 µg/ml PI for 15 min at room temperature. Hoechst can stain all of the cells in blue, while the dead cells were shown by PI nuclear staining in red. For each sample, about 500 cells were visualized, random captured and counted for cell viability based on the ratio of PI-positive to negative cells using an inverted fluorescent microscope (Nikon ECLIPSE TE2000-S).

**Figure 3:**
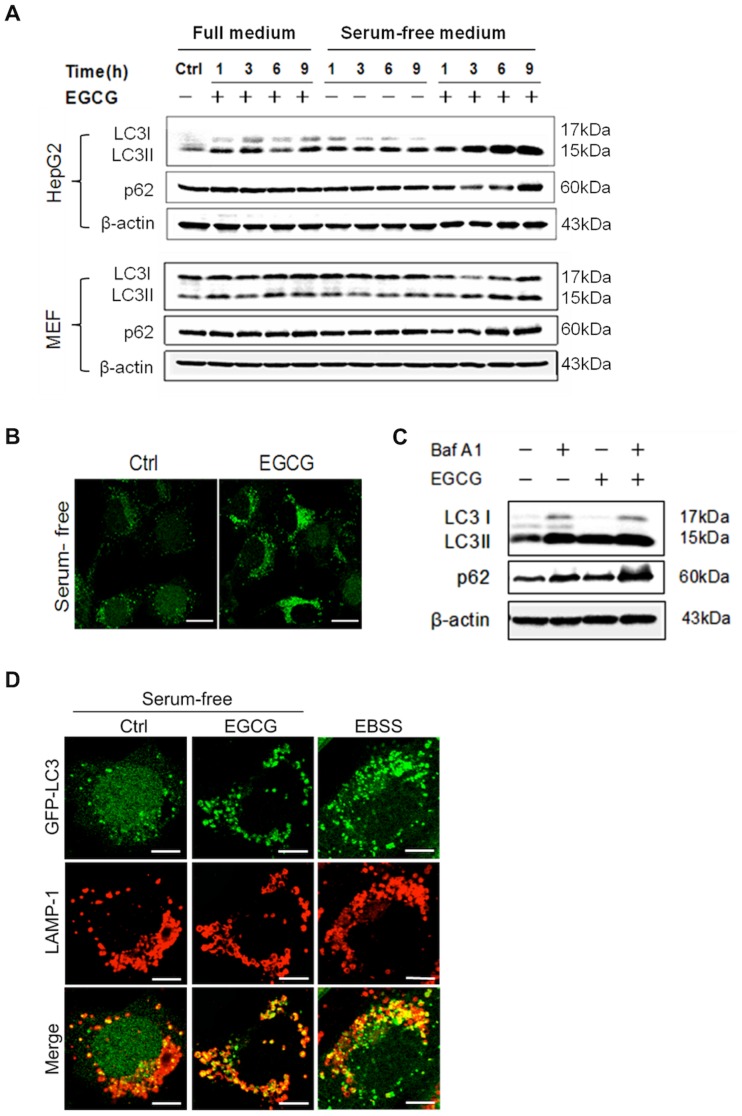
EGCG blocks autophagic flux. (A) EGCG increases LC3-II and p62 protein level. HepG2 and MEF cells were treated with EGCG (60 µM) for indicated durations in full medium or in serum-free medium as indicated. Cell lysates were collected for western blot. (B) EGCG increases the formation of GFP-LC3 puncta. MEF with stable expression of GFP-LC3 (m5–7 cells) were treated with or without 60 µM EGCG for 9 h in serum-free medium and analyzed by confocal microscopy (scale bar: 20 µm). (C) EGCG does not promote autohpagic flux. HepG2 cells were treated in serum-free medium with 60 µM EGCG, 50 nM Baf A1, or both for 9 hours. Cell lysates were collected and subject to western blot. (D) EGCG has no effect on autophagosome-lysosome fusion. MEF cells with stable expression of GFP-LC3 were cultured in serum-free medium for 9 h (as a control); treated with EGCG (60 µM×9 h) in serum-free medium; or cultured in EBSS medium for 2 h (as a positive control). Cells were then, stained LAPM-1 as described in Figure 2D. Cells were analyzed by confocal microscopy (scale bar: 10 µm).

### Oil Red O for Lipid Droplet Staining

Lipid droplet staining was performed using an established method [26]. Briefly, HepG2 cells were seeded to 24-well plate on a coverglass and cultured overnight. For the positive control, cells were treated with 1 mM oleic acid in serum-free DMEM containing 1% BSA. After treatment, cells were fixed with formaldehyde, and then stained using 0.5% Oil-Red-O in isopropanol for 30 min and nuclei were counter-stained with hematoxylin.

### Immunofluorescence Staining

In this study, the lysosome-associated membrane protein (LAMP-1) was examined by immunofluorescence staining, based on an established method [27]. Treated cells were fixed with 4% paraformaldehyde in PBS for 15 min at room temperature, and permeabilized with 0.01% saponin in PBS for 10 min. After blocking with 1% BSA in PBS for 30 min, cells were incubated with rat anti-mouse LAMP-1 (1D4B) primary antibody (Developmental Studies Hybridoma Bank) in a 1∶100 dilution overnight at 4°C, followed by Alexa Fluor 555 goat anti-rat secondary antibody (Invitrogen). The cells were examined using a confocal microscope (Olympus Fluoview FV1000) and representative cells were selected and photographed.

**Figure 4:**
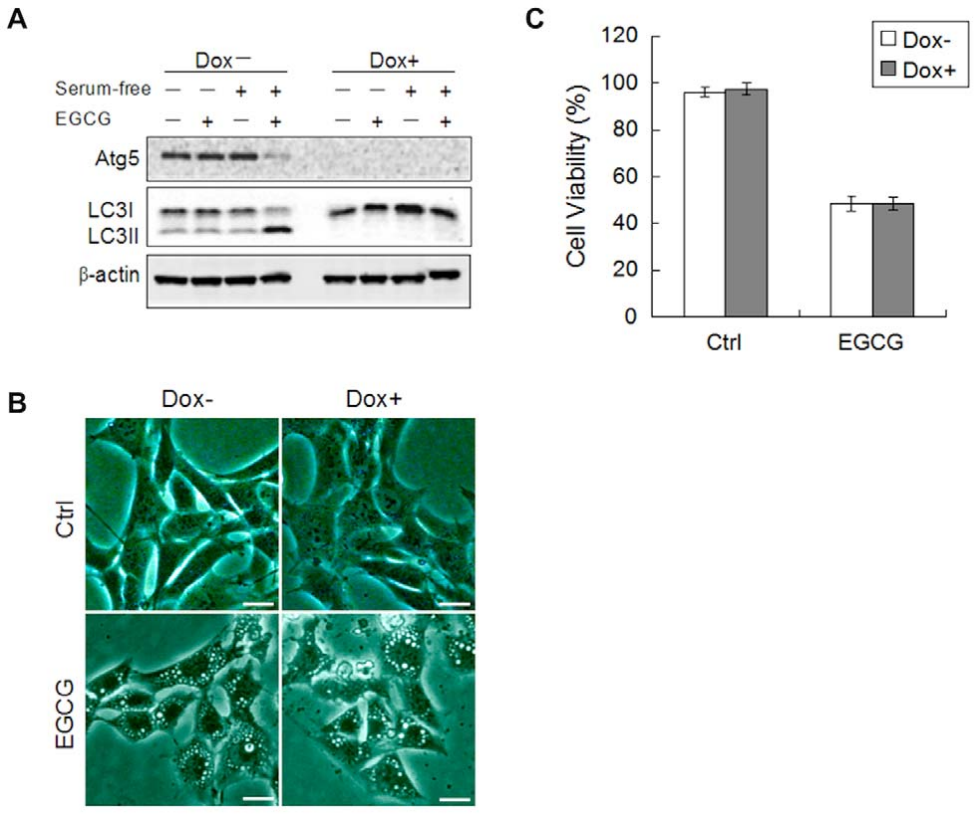
EGCG causes cell death independent of autophagy. (A) MEF cells with inducible deletion of Atg5 (m5–7 cells) were cultured with or without 10 ng/ml Dox for 4 days, then EGCG (60 µM×6 h), and cell lysates were collected and subject to western blot. (B) The formation of vacuoles is Atg5-independent. m5–7 cells as described in panel A were treated with EGCG (60 µM) for 12 h and observed under light microscopy (scale bar: 30 µm). (C) EGCG-induced cell death is independent of autophagy. m5–7 cells as described in panel A were treated with EGCG (60 µM) for 24 h. The cell viability was determined by Hoechst-PI double staining (n = 3, mean ± SD).

### Lysosomal Staining

Lysosomal staining was performed using Lyso-Tracker, a lysosomotropic probe (Invitrogen) [28]. The treated cells were incubated for 30 min at 37°C with 5 nM of Lyso-Tracker. The cells were examined using a confocal microscope and representative pictures were photographed.

### Preparation of Membrane Fraction by Digitonin Extraction

The method to separate the cytosolic fractions from membrane fractions was used as previously reported [29]. After designated treatments, cells were first suspended in the extraction buffer (250 mM sucrose, 20 mM HEPES pH 7.5, 10 mM KCl, 1.5 mM MgCl_2_, 1 mM EDTA, 1 mM EGTA, with the addition of with 250 µg/ml digitonin (Sigma) for 10 minutes on ice with repeated vortexing, followed by centrifugation (90 seconds, 13,000 rpm, 4°C). The resulted supernatant was then kept as the cytosolic fraction and pellet as the membrane fraction. This optimal concentration of digitonin in the extraction buffer was determined by which it gives efficient extraction of cytosolic proteins (analyzed by immunoblots using GAPDH as a marker), while still leaving lysosomal membranes intact (using cathepsin D as a marker).

### Western Blotting

At the end of the designated treatments, cells were lysed in whole cell lysis buffer (62.5 mM Tris-HCl at pH 6.8, 20% glycerol, 2% SDS, 2 mM DTT, 100 µM PMSF and proteinase inhibitor cocktail). Samples with equal amount of proteins were subjected to SDS-PAGE and transferred to a polyvinylidene fluoride (PVDF) membrane (Bio-Rad). After blocking with 5% non-fat milk, probed with designated primary and secondary antibodies. The membrane was developed with the enhanced chemiluminescence method (Thermo Scientific) and visualized with the Kodak Image Station 4000R (Kodak).

**Figure 5:**
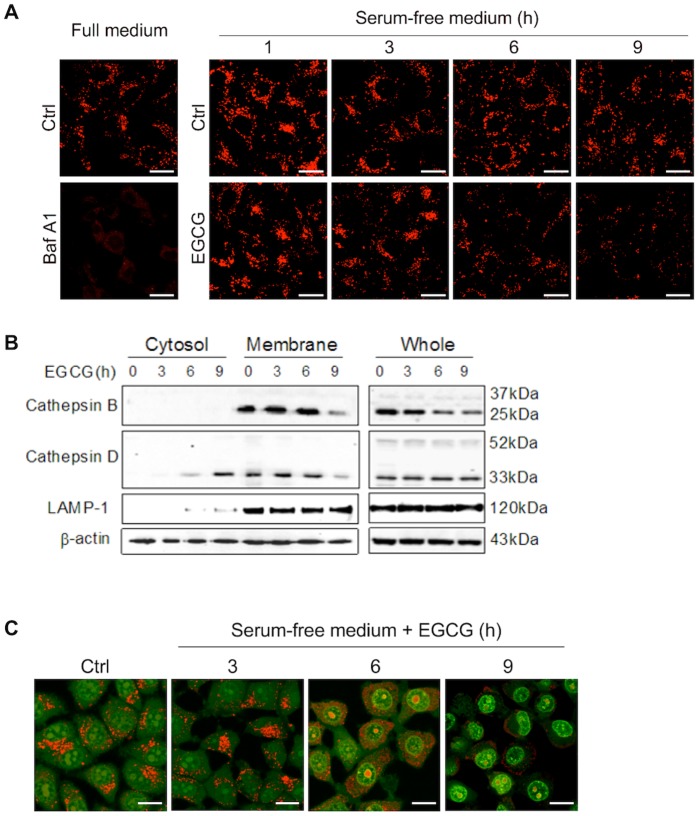
EGCG induces LMP. (A) Effect of EGCG on intracellular acidic compartments. HepG2 cells were with EGCG (60 µM) for indicated durations or with Baf A1 (50 nM) for 6 h. The acidic compartments were labeled by 5 nM Lyo-Tracker Red and examined by confocal (scale bar: 20 µm). (B) EGCG induces the leakage of cathepsins from lysosome to cytosol. Cell fractionation was performed to separate lysosomal and cytosolic fractions in HepG2 treated with 60 µM EGCG in serum-free medium as indicated. Cathepsin D was detected by western blotting in the different fractions and whole cell lysates. LAMP-1 was used as a marker for lysosome. (C) EGCG causes lysosomal neutralization and cytosolic acidification. HepG2 cells were treated with 60 µM EGCG as indicated in serum-free medium, followed by staining with 5 µg/ml acridine orange (AO) for 30 min and analyzed by confocal (scale bar: 20 µm).

### Acridine Orange (AO) Staining

AO is a metachromatic and weak base membrane-permeant fluorescent dye, whose fluorescence emission is concentration-dependent, from red at high concentrations (in lysosomes) to green at low concentrations (in the cytosol), with yellow as intermediate in some conditions [19]. In our experiments, cells were seeded to a coverglass slide chamber at 3×10^4 ^per well. After the designated treatments, they were loaded with AO (5 µg/ml) at 37°C for 15 min, rinsed, then examined under confocal microscope with excitation wavelength at 488 nm, while two separate emission bands (505–570 nm and 615–754 nm) were simultaneously collected.

### Detection of Reactive Oxygen Species

Analysis of intracellular ROS production was conducted using an established method [30]. Briefly, HepG2 cells with indicated treatments were incubated with 10 µM CM-H_2_DCFDA at 37°C for 15 and analyzed under a fluorescence microscope. Representative pictures of time courses were selected and photographed.

### Statistical Analysis

The numeric data were presented as mean ± SD from at least 3 independent experiments. Statistical analysis was calculated using Student’s *t*-test (two-tailed distribution, unequal variance).

**Figure 6:**
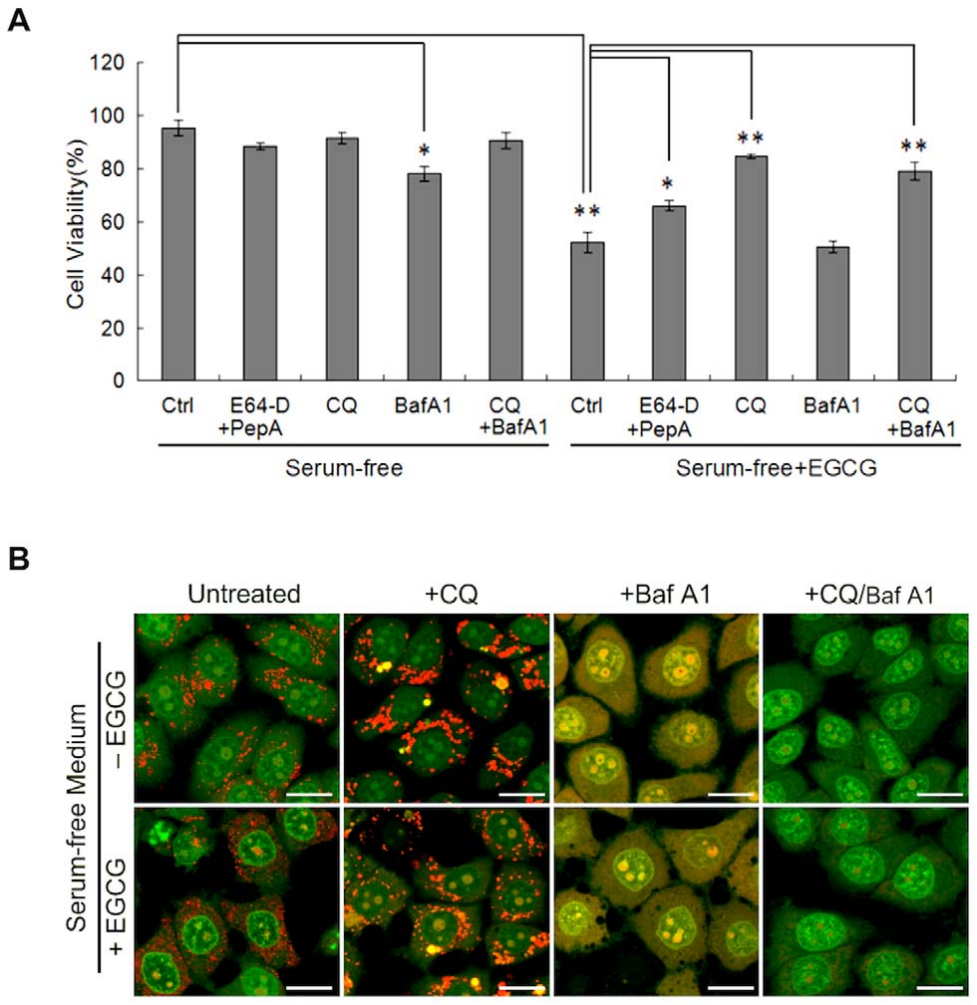
EGCG induces cell death via LMP. (A) Different lysosome inhibitors present different effects on EGCG-induced cell death. HepG2 cells were treated for EGCG (60 µM×24 h) in serum-free medium in the absence or presence of various inhibitors, including E64-D (10 µg/ml) + pepstatin A (10 µg/ml), chloroquine (CQ, 25 µM), Baf A1 (50 nM). The cell viability was determined by Hoechst-PI double staining (n = 3, mean ± SD). The *p* values were determined using Student's *t*-test (*P<0.05, **P<0.005). (B) CQ, but not Baf A1 blocks the cytosolic acidification induced by EGCG. HepG2 cells were with EGCG (60 µM), CQ (25 µM) or Baf A1 (50 nM) in serum-free medium for 6 h followed by staining with 5 µg/ml AO for 30 min and analyzed by confocal (scale bar: 20 µm).

**Figure 7:**
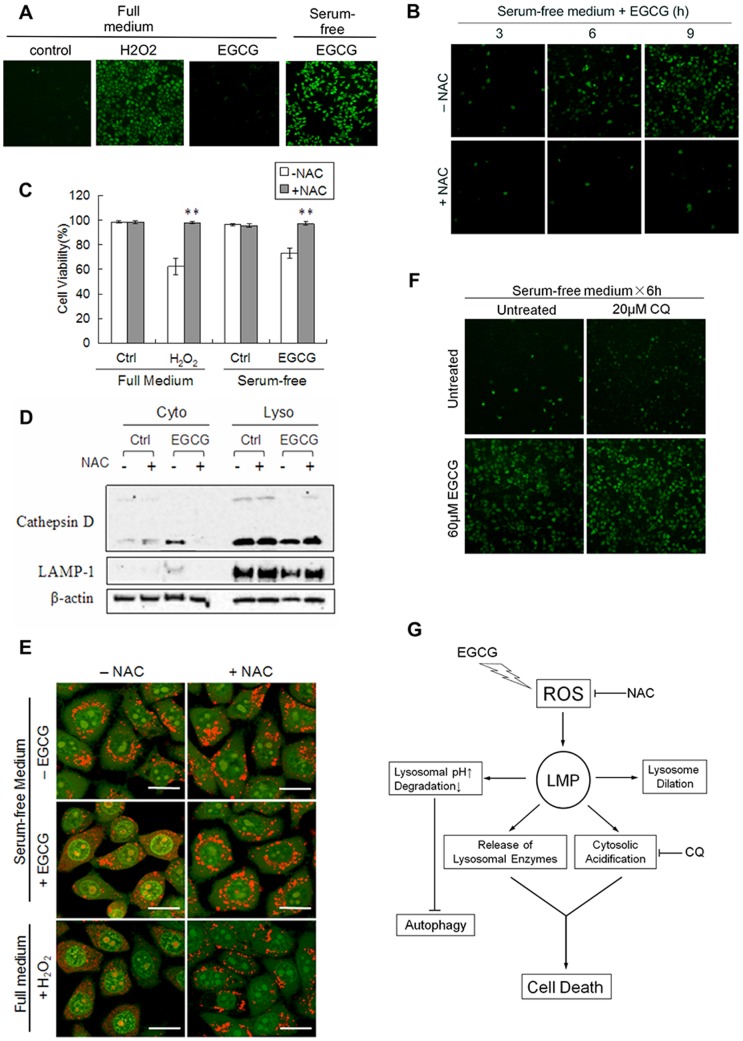
ROS mediates LMP and cell death induced by EGCG. (A) EGCG induces intracellular ROS production. HepG2 cells were treated with EGCG (60 µM) for 6 h in full medium or in serum-free medium Treatment with H_2_O_2_ (200 µM) for 3 h was used as a positive control. The intracellular ROS was detected by CM-H_2_DCFDA and analyzed under a fluorescence microscope. (B) NAC prevents ROS formation induced by EGCG in serum-free medium. Cells were treated with EGCG (60 µM×6 h) in the absence or presence of N-acetylcysteine (NAC, 5 mM). (C) Protection by NAC of EGCG-induced cell death. HepG2 cells were treated by EGCG (60 µM) or H_2_O_2_ (200 µM) as shown for 12 h in the absence or presence of 5 mM NAC. The cell viability was determined by Hoechst-PI double staining (n = 3, mean ± SD). **P<0.005 in comparison to the group without NAC (Student's *t*-test). (D) NAC prevents cathepsin D translocation caused by EGCG. HepG2 cells were treated with EGCG (60 µM×6 h) with or without 5 mM NAC. Both the lysosomal and cytosolic fractions were analysed by western blot. (E) NAC prevents EGCG-induced cytosolic acidification. HepG2 cells were treated with EGCG (60 µM×6 h) or H_2_O_2_ (200 µM×3 h) with or without 5 mM NAC. Cells were then stained with AO for 30 min and analyzed by confocal (scale bar: 20 µm). (F) CQ fails to affect ROS production induced by EGCG. HepG2 cells were cultured in serum-free medium for 1 h, then added 60 µM EGCG, with or without the presence of 20 µM CQ for 6 h. (G) Illustration for the mechanisms underlying EGCG-mediated caspase-independent cell death, involving ROS and LMP.

## Results

### Serum Starvation Enhances EGCG-induced Cell Death

EGCG has been previously reported to induce apoptotic cell death in a variety of human cancer cell lines [31]. One surprising finding in the course of our study is that the presence of 10% serum has a major impact on the cytotoxicity of EGCG tested. As shown in Figure 1A, HepG2 cells were largely resistant to EGCG in full medium as high as 240 µM cultured for up to 36 hrs, while EGCG is able to markedly reduce the cell viability of HepG2 cells when treated in serum-free medium. Similar effects were observed in other human cancer cell lines such as HeLa cells (data not shown). Therefore, our results suggest that serum starvation is able to promote EGCG-induced cell death.

Next we set to determine the form of cell death induced by EGCG in serum-free medium. To do this, we first examined the morphological changes of the nuclei with Hoechst staining. As shown in Figure 1B, the nuclei in cells treated with EGCG did not show any obvious changes, while evident nuclear condensation was observed in cells treated with TNFα and CHX, as a positive control for typical apoptotic cell death. Moreover, z-VAD-fmk, a general caspase inhibitor failed to protect against EGCG-induced cell death, while it completely prevented cell death induced by TNFα and CHX (Figure 1C). Consistently, there was no activated caspase-3 (17 kDa) or cleaved PARP (89 kDa) detected by western blotting (Figure 1D) in HepG2 cells treated with EGCG. In contrast, such apoptosis hallmarks were found in cells treated with TNFα and CHX and prevented by z-VAD-fmk. Taken together, it is clearly that EGCG induces cell death under serum-free conditions is likely to be caspase-independent non-apoptotic cell death.

### EGCG Triggers Cytosolic Vacuolization Due to Lysosome Dilation in Serum-free Medium

One morphological feature observable under the light microscope was cytosolic vacuolization in the cells treated with EGCG in serum-free medium and the time and dose-dependent changes were presented in Figure 2A. Notably, no such vacuolization was found in cells treated with EGCG in full medium (Figure 2B). Similar results were also found in HeLa cells with the same treatment (data not shown). Such vacuolization was also visible in cells stained with hematoxylin, a nuclear dye (Figure 2B). Another visible morphological feature in cells treated with the highest concentration of EGCG in serum-free medium is the lost of cytosolic content, with increased cell size (Figure 2B).

Considering the close relationship between vacuolization and cell death, we then tried to find out the nature sources of the cytosolic vacuoles. The vacuolization was reported to be induced by a wide range of inductive stimuli and involved many different organelles and structures, such as endoplasmic reticulum (ER), Golgi, and lysosome [32]. In our study, we first excluded the possibility that the vacuoles induced by EGCG are lipid drops, as they are negative to staining with Oil Red O (Figure 2C), in contrast to cells treated with OA that were used a positive control. Moreover, such vacuoles were also not associated with ER or Golgi based on immunostaining of respective marker proteins (data not shown). Interestingly, we found that such vacuoles well co-localize with lysosomal membrane protein LAMP-1 (Figure 2D), suggesting that the vacuoles were closely associated with lysosomes.

### EGCG Blocks Autophagic Flux

The fact that serum starvation enhances EGCG-induced cell death prompts us to examine the role of autophagy in EGCG-mediated cell death. It has been well established that starvation induces autophagy and autophagy is closely implicated in the cell death process, either as a pro-survival or pro-death mechanism [33]. To test this, we first examined the autophagy level in cells treated with EGCG, by analyzing the LC3-II level, a widely used marker for autophagosomes [34]. As shown in Figure 3A, there was significant increase of LC3-II in EGCG-treated cells, in both HepG2 and MEF, in serum-free medium, but not in full medium. Similarly, there was significant increase of GFP-LC3 puncta in MEF with stable expression of GFP-LC3 (Figure 3B). In this study, we used a number of approaches to examine whether the increased autophagic markers observed in EGCG-treated cells is due to increased level of autophagic flux or blockage of autophagosome maturation or degradation at the late stage. First, we used bafilomycin A1 (Baf A1), an inhibitor of lysosomal V-ATPase, to block autophagosome-lysosome fusion [35]. Notably, Baf A1 failed to further increase LC3-II levels (Figure 3C). Second, we examined the change of p62 protein level. It is known that p62 is selectively incorporated into autophagosomes through direct binding to LC3 and efficiently degraded by autophagy [34]. We observed evident accumulation of p62 protein in both HepG2 and MEF treated with EGCG (Figure 3A, 3C). Finally, we found that EGCG treatment significantly enhanced the co-localization of autophagosome (marked by GFP-LC3) and lysosome (marked by LAMP-1), comparing to the control cells with serum-starvation (Figure 3D). Taken together, all data described above clearly indicate that EGCG is able to inhibit the autophagic flux by blocking the late stage autolysosome degradation.

### EGCG-mediated Cell Death is Independent of Autophagy

After establishing the inhibitory effect of EGCG on autophagy, we aimed to test whether the disrupted autophagic flux contributes to the EGCG-induced cell death. Here we utilized the m5–7 cells with inducible Atg5 deletion [24]. As shown in Figure 4A, addition of doxycycline (Dox) eliminated Atg5 and change of LC3-II. Interestingly, deletion of Atg5 had no effect on cytosolic vacuolization induced by EGCG (Figure 4B), and eventually cell death (Figure 4C). Therefore, it is believed that EGCG-mediated cell death is independent of Atg5 or autophagy.

### EGCG Induces Lysosomal Membrane Permeabilization (LMP)

Earlier data found in this study indicate the possibility of lysosomal damage caused by EGCG. In order to further examine the changes of lysosomal function caused by EGCG, we first used Lyso-Tracker Red (LTR) to label and track the acidic intracellular compartments (lysosomes) in live cells. As shown in Figure 5A, addition of EGCG markedly reduced the fluorescence in a time-dependent manner, indicating a reduction of intracellular acidic components caused by EGCG treatment. As expected, treatment of Baf A1 completely abolished the LTR staining due to abolished lysosomal acidification.

As an acidotropic probe, the decreased LTR fluorescence may not only reflect an increase in lysosomal pH, but also suggest the possibility of lysosomal membrane permeabilization (LMP) [19]. To determine whether EGCG could trigger the LMP, the distribution of cathepsin B and cathepsin D, two key lysosomal enzymes, was compared by immunoblots between the cytosolic and membrane fractions. As shown in Figure 5B, there was a time-dependent increase of a mature form of cathepsin D in cytosolic extraction in cells treated with EGCG, corresponding to the decrease in the membrane fraction. Such a temporal pattern is in fact consistent with the reduction of LTR staining shown in Figure 5A, indicating a leakage of lysosomal content into the cytosol.

EGCG-induced LMP was further confirmed by staining of acridine orange (AO), a lysosomotropic metachromatic fluorochrome, which normally fluoresces red inside the lysosomes where it is highly concentrated, and weakly green in the cytosol with a lower concentration [19]. As shown in Figure 5C, EGCG treatment time-dependently led to loss of red puncta in lysosomes, and increase of diffused red fluorescence in cytosol, with a consistent temporal pattern with LTR staining and leakage of cathepsin D shown earlier. Such observations thus suggest that EGCG causes the rupture of lysosome, which then triggers a release of acidic contents from the lysosomal lumen to cytosol, with reduction of lysosomal acidification and dramatic increase of cytosolic acidification. Noteworthy, the nucleic green fluorescence was also enhanced over time, indicating nuclei acidification with prolonged treatment of EGCG (Figure 5C). The importance of such changes in EGCG-induced cell death remains to be further studied.

### EGCG Induces LMP-dependent Cell Death

Up to this point, we have shown that EGCG induces caspase-independent cell death, independently of autophagy. More importantly, we have identified lysosome as the main target of EGCG, evidenced by lysosomal membrane permeabilization, loss of cytosolic acidification and lysosome dilatation. Here we aimed to test whether the impairment of lysosomes plays a causative role in EGCG-induced cell death. First, we used several different lysosome inhibitors and among them, chloroquine (CQ), a lysosomotropic agent, offered the most effective protection against EGCG-induced cell death (Figure 6A). Two cathepsin inhibitors (E64-D and pepstatin A) were also effective, although to a much lesser extent. Interestingly, Baf A1 failed to protect against EGCG-induced death, and treatment with Baf A1 alone in serum-free medium is also slightly cytotoxic (Figure 6A).

Since both CQ and Baf A1 are able to suppress lysosomal function, their distinct effects on EGCG-induced cell death are indeed intriguing and deserve further investigation. Considering EGCG-mediated LMP is believed to be critical in its cytotoxicity, we compared the effects of CQ and Baf A1 on EGCG-induced LMP by examining the changes of cytosolic acidification measured by AO staining. As shown in Figure 6B, CQ was able to reverse the lysosomal membrane damage caused by EGCG, by restoring the lysosomal red staining. In contrast, Baf A1 caused lost of lysosomal red staining and increase of cytosolic acidification evidenced by yellow staining of the cell. Such changes are consistent with an early report that Baf A1 was able to cause intracellular acidification [36]. Interestingly, CQ was able to reverse the cytosolic acidification, but it failed to restore the lysosomal pH in cells treated with Baf A1 (Figure 6B). Such findings could help to explain the fact that CQ is still effective in protecting against EGCG-mediated cell death in the presence of Baf A1 (Figure 6A). On the other hand, it is intriguing that CQ failed to neutralize lysosomal pH in both cells with or without EGCG exposure (Figure 6B). Although hard to explain, our data are indeed consistent with a very recent report in which treatment with CQ (from 10 to 100 µM) for 24 hrs enhanced AO lysosomal staining [37]. Together, our data suggest that LMP-induced cytosolic acidification is a critical step in EGCG-caused cell death, a process could be inhibited by prolonged treatment of CQ, but not by Baf A1.

### ROS and Oxidative Stress Trigger EGCG-mediated LMP and Cell Death

As mentioned earlier, EGCG is unstable in culture medium and will undergo auto-oxidative reactions resulting in substantial amount of ROS production [10], [11], [38]. Here we explored whether such ROS are involved in EGCG-induced cell death. In our experiments, we first found early production of intracellular ROS after EGCG treatment under serum-free medium (Figure 7A), but not in full medium. Addition of an antioxidant N-acetylcysteine (NAC) almost completely suppressed ROS formation (Figure 7B). As a result, NAC was able to prevent EGCG-mediated cell death (Figure 7C). The effect of NAC on H_2_O_2_-induced cell death was used as a positive control (Figure 7C). The above data thus indicate that the increased ROS play an important role in EGCG-induced cell death. As ROS have been reported to be among the principal inducers of LMP [19], here we further investigated whether ROS are responsible for LMP observed in EGCG-treated cells. As shown in Figure 7D, addition of NAC prevented the leakage of cathepsin D from lysosome. Moreover, using AO staining, we found that either EGCG or H_2_O_2-_induced cytosolic acidification can be blocked by NAC (Figure 7E). Consistently, NAC also protected for formation of cytosolic vacuolization in ECGC-treated cells (data not show). Finally, we provided evidence that CQ was unable to affect ROS level in cells treated EGCG (Figure 7F). All these results clearly reveal that EGCG-mediated ROS formation is the upstream mechanism contributing to LMP and cytosolic acidification, and eventually cell death.

## Discussion

At present the mechanisms for the anti-cancer function of EGCG has been extensively studied. It is known that EGCG is capable of targeting multiple cellular pathways and damaging various subcellular organelles to suppress cell proliferation and induce cell death, while its effects on lysosome have not been reported [31]. In this study, we provided compelling evidence showing EGCG-induced LMP as the key mechanism in EGCG-mediated cell death. Moreover, we identified the pro-death function of ROS and oxidative stress upstream of LMP. Therefore, data from this study reveal a novel mode of action for the cytotoxic activity of EGCG: an early production of ROS causes destabilization of lysosomal membrane, leading to release of lysosomal enzymes, the acidification of cytosol, and the dilation of lysosomes, and ultimately, these alterations commit cells to death via a non-apoptotic pathway.

One of the key findings in this study is the critical role of LMP in EGCG-induced cell death. Onset of LMP has been closely associated with cell death, both apoptosis and non-apoptotic cell death, largely depending on the extent of lysosomal membrane damage and other cellular context [17]. The direct consequence of LMP is the leakage of lysosomal hydrolytic enzymes that could set off indiscriminate degradation of cellular components and trigger the cell death pathways [19], [21]. In this study, EGCG-mediated LMP was clearly demonstrated via evident lysosomal dilation (increased cytosolic vacuolization, Figure 2), cytosolic translocation of lysosomal enzyme cathepsins (Figure 5B), increase of lysosomal pH (by LTR staining, Figure 5A), and increased cytosolic acidification (by AO staining, Figure 5C), eventually leading to non-apoptotic cell death in human cancer cells. Notably, prevention of LMP by either CQ or NAC almost completely abolished EGCG-induced cell death (Figure 6A, 7B), suggesting that EGCG-induced LMP is both necessary and sufficient for non-apoptotic cell death. If LMP occurs selectively, lysosomal proteases which may be implicated in cell death are commonly the cathepsins that have low-molecular weight and may remain active at neutral pH [18]. In that case, the inhibition of a single cathepsin isoform is sufficient to retard cell death [39]. Nevertheless, our experiments show that the major cathepsin inhibitors cannot efficiently block EGCG-induced cell death, though activated cathepsin D is detected in cytosol. In addition, the significant reduction of LRT staining prompts that the cytosol-lysosome pH gradient may be collapsed by EGCG treatment. Another notable finding is although we prove the cytosolic vacuoles are formed by the dilated lysosomes, most of larger vacuoles actually are not labeled by LRT (data not show), but can be stained by LAMP-1. This could be explained that larger lysosomes are more susceptible to rupture than small lysosomes. Collectively, these data point out that EGCG-induced LMP leads to a massive impairment of lysosomes. Therefore, understanding the involvement of LMP in EGCG-induced cell death shed new lights on the molecular mechanisms underlying the anti-cancer function of EGCG.

One major consequence of LMP is cytosolic acidification. Generally, cytosolic pH is tightly controlled within a narrow range for the maintenance of normal cell function. Thus, the severe alteration of cytosolic pH is known to disturb the cellular homeostasis and trigger a cascade of molecular events which finally leads to cell death [40]. In the case of receptor or drug-induced apoptosis, the acidification has been shown to occur early during the process in some model systems, or to be a later caspase-dependent event in others [41], [42]. Cytosolic acidification has also been implicated in necrotic cell death, based on a report in which intracellular pH was found to an important modulator of necrosis in *C. elegans* [43]. One intriguing finding from this study is that CQ is capable of effectively protecting against ECGC-induced cell death, whereas Baf A1 fails to offer any protection. With the help of AO staining, we infer the possible explanation for the differences between CQ and Baf A1 to their different effects on cytosolic acidification: CQ, but not Baf A1, prevents cytosolic acidification (Figure 6B). Such observations are also consistent with an earlier report in which Baf A1 is found to cause cytosolic acidification and induce apoptosis [36]. As the enzymatic activity of many cathepsins such as cathepsin B, D and L are pH dependent, prevention of cytosolic acidification and neutralization of the cytosolic pH would then offer significant protection by reducing the enzymatic activity of those lysosomal hydrolases that leak into cytosol.

After establishing the critical role of LMP in EGCG-induced cell death, we further identified the higher level of ROS and oxidative stress as the upstream mechanisms. The generation of ROS by EGCG in cell cultured system has been reported previously [10], [11]. The cytotoxic effects of EGCG, such as inhibition of growth and induction of apoptosis, are partially attributed to this pro-oxidative action which produces hydrogen peroxide (H_2_O_2_) in both culture medium and intracellularly [44]. Although at this stage the exact mechanisms for EGG-mediated ROS formation is not clear, it seems to be a common feature for many flavonoids such as luteolin to promote ROS formation [45]. In our study, higher level of intracellular ROS was found in EGCG-treated cells, an event preceding the onset of LMP and cell death (Figure 7). More importantly, an antioxidant NAC can block LMP and prevent the subsequent cell death (Figure 7). This result is consistent with the common understanding that ROS are one of the well-established inducers of LMP and ROS and oxidative stress are often implicated as the underlying mechanism contributing to lysosomal destabilization observed in cells treated with a wide range of chemical reagents [19]. It is believed that lysosomes are susceptible to ROS due to the deficiency of antioxidant enzymes in lysosomes, thus, the produced ROS such as H_2_O_2_ can easily diffuse into lysosomes to cause impairment of the lysosomal membrane via oxidative damage such as lipid peroxidation [46].

Another interesting aspect of our study is the fact that serum starvation markedly enhanced EGCG-mediated cell death (Figure 1). Since starvation is known to induce autophagy and lysosomal-dependent degradation is an integral part of the autophagic process [47], it is thus of interest to determine the involvement of autophagy in EGCG-mediated cell death. First, by using a range of assays, including of LC3 turnover assays, examination of endogenous autophagy substrate p62 accumulation, and analysis with the co-localization of GFP-LC3 and LAMP-1 [34], we have presented sufficient evidence that EGCG primarily acts to block autophagic flux, most probably via the disruption of lysosomal function as described earlier. Second, EGCG-induced lysosomal damage and cell death was found to independent of autophagy or Atg5 (Figure 4). At present, the role of autophagy in cell death is a highly controversial topic. Under many of the stress conditions, autophagy is generally playing a pro-survival function [48], while the pro-death function of autophagy has also been widely reported, either via promotion of apoptosis or via so-called “autophagic cell death” [49]. At present, the involvement of autophagy in LMP and lysosome-dependent cell death remains poorly understood. In hepatocytes treated with interferon-gamma (IFN-γ) and Concanavalin A (Con A), autophagy was found to promote LMP-mediated necrotic cell death [50]. Similarly, LMP has been found to contribute to autophagic cell death under oxidative stress [51]. Therefore, functional relationship between autophagy and LMP in control of cell death deserves further investigations.

As discussed above, since autophagy is found to be independent of cell death induced by EGCG (Figure 4), the more reasonable explanation of our data is that it is not serum starvation that helps killing the cells, rather it is the presence of serum that markedly reduces the cytotoxicity of EGCG. In fact we have evidence that addition of BSA into the FBS-free medium offered dose-dependent protection against EGCG-mediated cell death (data not shown). One possible mechanism for the protective effect of serum or BSA is the H-bonding capacity of EGCG that enables EGCG binding to proteins, thus reduces the bioavailability of EGCG to the cultured cells, which eventually reducing the cytotoxicity of EGCG [52]. Our ongoing study is aiming to reveal how the binding of EGCG to extracellular proteins would affect the biological function of EGCG.

Taken together, here we establish a novel mechanism underlying EGCG-mediated non-apoptotic cell death via ROS and LMP, but independent of autophagy (Figure 7G). We believe that our findings provide important information for understanding the molecular mechanisms underlying EGCG-mediated cell death and anti-cancer function.
